# Dynamics of HBV cccDNA expression and transcription in different cell growth phase

**DOI:** 10.1186/1423-0127-18-96

**Published:** 2011-12-30

**Authors:** Chin-Liew Chong, Mong-Liang Chen, Yi-Chieh Wu, Kuen-Nan Tsai, Chien-Chiao Huang, Cheng-po Hu, King-Song Jeng, Yu-Chi Chou, Chungming Chang

**Affiliations:** 1 Institute of Microbiology and Immunology, National Yang-Ming University, 155, Sec.2, Linong Street, Taipei, 112, Taiwan; 2 Institute of Molecular and Genomic Medicine, National Health Research Institutes, 35, Keyan Road, Zhunan Town, Miaoli, 350, Taiwan; 3 Center for Molecular Medicine, China Medical University and Hospital, 2, Yude Road, Taichung, 40447, Taiwan; 4 Institute of Molecular Medicine, National Tsing Hua University, 101, Sec.2, Kuang-Fu Road, Hsinchu, 30013, Taiwan; 5 Department of Life Science, Tunghai University, 181, Sec.3, Taichung Port Road, Taichung, 40704, Taiwan; 6 Institute of Molecular Biology, Academia Sinica, 128, Sec. 2, Academia Road, Nankang, Taipei, 115, Taiwan

**Keywords:** HBV, cccDNA, viral replication, cell proliferation, growth confluency

## Abstract

**Background:**

The covalently closed-circular DNA (cccDNA) of hepatitis B virus (HBV) is associated with viral persistence in HBV-infected hepatocytes. However, the regulation of cccDNA and its transcription in the host cells at different growth stages is not well understood.

**Methods:**

We took advantages of a stably HBV-producing cell line, 1.3ES2, and examine the dynamic changes of HBV cccDNA, viral transcripts, and viral replication intermediates in different cellular growth stages.

**Results:**

In this study, we showed that cccDNA increased suddenly in the initial proliferation phase of cell growth, probably attributable to its nuclear replenishment by intracellular nucleocapsids. The amount of cccDNA then decreased dramatically in the cells during their exponential proliferation similar to the loss of extrachromosomal plasmid DNA during cell division, after which it accumulated gradually while the host cells grew to confluency. We found that cccDNA was reduced in dividing cells and could be removed when proliferating cells were subjected to long term of lamivudine (3TC) treatment. The amounts of viral replicative intermediates were rapidly reduced in these proliferating cells and were significantly increased after cells reaching confluency. The expression levels of viral transcripts were increased in parallel with the elevated expression of hepatic transcription factors (HNF4α, CEBPα, PPARα, etc.) during cell growth confluency. The HBV transcripts were transcribed from both integrated viral genome and cccDNA, however the transcriptional abilities of cccDNA was less efficient then that from integrated viral genome in all cell growth stages. We also noted increases in the accumulation of intracellular viral particles and the secretion of mature virions as the cells reached confluency and ceased to grow.

**Conclusions:**

Based on the dynamics of HBV replication, we propose that HBV replication is modulated differently in the different stages of cell growth, and can be divided into three phases (initial proliferation phase, exponential proliferation phase and growth confluency phase) according to the cell growth curve. The regulation of cccDNA in different cell growth phase and its importance regarding HBV replication are discussed.

## Background

Infection with hepatitis B virus (HBV), which can cause acute and chronic liver diseases, remains one of the most serious viral infections in humans. Approximately 400 million people worldwide suffer from chronic hepatitis B (CHB) infection, and many of them have a high risk of developing cirrhosis or hepatocellular carcinoma [1,2]. In CHB patients, a pool of covalently closed circular DNA (cccDNA), generated from the relaxed-circle (RC) form of viral DNA, is maintained in the nuclei of infected hepatocytes and acts as the template for viral gene expression [3]. Within infected cells, the pregenomic RNA (pgRNA) is transcribed from cccDNA and reverse transcribed into RC form of viral DNA in the viral capsids [4]. The mature capsids either are secreted from the cells or re-enter the nucleus to replenish the cccDNA pool [5,6].

In addition to its crucial role in HBV life cycle, the existence of cccDNA interferes with the outcomes of clinical antiviral therapy. For example, lamivudine (3TC), an antiviral nucleoside analogue which inhibit viral polymerase activity, effectively inhibits HBV replication and eliminate the HBV virion from the blood of patients. However, the cessation of drug treatment results in the rapid reappearance of HBV in the serum [7,8]. In vitro studies have shown that the persistence of cccDNA is responsible for the recurrence of HBV infection [9]. Several studies have demonstrated that cccDNA is a very stable molecule. After treatment with antiviral drugs, the half-life of cccDNA was reported to range from 33 to 57 days in these hepadnaviruese-infected woodchucks and ducks [10,11]. Traces of cccDNA persisted indefinitely in the livers of HBV-infected chimpanzees and provided a continuous antigenic stimulus that conferred lifelong immunity [12]. Therefore, the elimination of cccDNA from infected cells, to achieve viral clearance, has become a major issue in the treatment of chronic HBV infection.

The regulatory mechanisms involved in the clearances of cccDNA pool are critical, but not well understood, processes during curing of chronic and acute HBV infection. It was generally believed that the clearance of cccDNA is mediated by the cellular immune response against HBV infection, which acts by: (a) the noncytopathic inhibitory effect of cytokines, which reduce the RC DNA precursors of cccDNA [13,14]; (b) the cytopathic effect of the cytotoxic T-lymphocyte (CTL) response, which destroys the infected hepatocytes; and (c) the dilution effect achieved with the compensatory proliferation of the hepatocytes (mitotic loss) [11], which partitions the cccDNA during cell division [15,16]. Among these, the antiviral effects of cytokines and CTL have been extensively investigated. However, the dilution effect and its relationship with the dynamics of cccDNA pool as well as HBV replication have not been examined closely.

Several lines of evidence have suggested that HBV replication is highly dependent on the growth status of hepatocytes. Clinical specimens show low levels of intrahepatic or serum-associated HBV during severe acute hepatitis at the time of active liver regeneration and inflammation, which represents a stage of rapid cell growth [17]. Whereas, high levels of intrahepatic HBV replication are observed in immunosuppressed patients or neonates with normal or near-normal hepatic histology, in which the hepatocytes remain in the quiescent stage [18]. Cell-cycle analysis has shown a correlation between elevated HBV replication and quiescent hepatocytes [17,19]. Similar phenomena have also been observed in an HBV-transfected hepatoblastoma-derived cell line, which showed increased viral production during the cell growth confluency [4,20]. Moreover, HBV replication and viral mRNA synthesis are significantly reduced in the proliferative stage [21,22].

In this study, we took advantages of a stably HBV-producing cell line, 1.3ES2, to determine the dynamic changes of HBV cccDNA, viral transcripts, and viral replication intermediates in different cellular growth stages. Based on the status of cell proliferation, we purpose a three-phase scenario (initial proliferation phase, exponential proliferation phase, and growth confluency phase) to describe the dynamic expression of viral cccDNA and its association with HBV replication. Furthermore, our findings also raise the possibility that facilitating hepatocyte regeneration may interfere with cccDNA metabolism and contribute to viral elimination.

## Methods

### Cell culture

The 1.3ES2 cell line is derived from HepG2 cells (a differentiated hepatoblastoma cell line) and contains one integrated copy of the HBV genome [23]. In this study, the cells were propagated in Dulbecco's modified Eagle's medium (DMEM) (Gibco Laboratories, Grand Island, NY) supplemented with 10% fetal bovine serum, 100 IU/ml penicillin, 100 μg/ml streptomycin, 2 mM L-glutamine, and 100 μM non-essential amino acid. The cells were grown at 37°C in a 5% CO_2 _incubator. In each experiment, the culture medium was changed every three days. Lamivudine (3TC) was added to a final concentration of 20 μM for the experiment in which 3TC was used as an inhibitor of HBV replication. A hemocytometer was used for microscopic cell counts. The cell samples collected at each time points were appropriately diluted with GKNP (0.1% glucose, 0.04% KCl, 0.8% NaCl, 0.006% KH_2_PO_4_, 0.01% phenol red, pH 7.4) containing Trypan Blue. All counts were made in triplicate.

### Hirt extraction of cccDNA and Southern blot analysis

To isolate cccDNA from 1.3ES2 cells, we used a previously described procedure, with modifications [23]. The cells with equal cell number were washed twice with ice-cold GKNP, and the residual washing solution was then removed as completely as possible. The cells were lysed by the addition of 3 ml of Hirt solution (0.6% SDS, 10 mM EDTA, 10 mM Tris-HCl, pH 7.5) for 5 min. After complete lysis of the cells, 750 μl of 5 M NaCl was added to the cell lysate and mixed gently. After the whole mixture had been incubated on ice overnight, the insoluble components were pelleted by centrifuging at 3, 000 × g for 15 min at 4°C. The supernatant, which contained the cccDNA, was extracted twice with phenol and once with phenol-chloroform, and then precipitated with the addition of two volumes of absolute ethanol. The precipitates prepared from equal amount of cells were dissolved with water and then heated at 85°C for 5 min to denature the DNA. After EcoRI or XhoI digestion, these DNA samples were loaded into agarose gel and separated by electrophoresis. The agarose gel was soaked twice in denaturing buffer (0.5 M NaOH, 1.5 M NaCl) for 15 min each and then neutralized with neutralizing buffer (1.5 M NaCl, 10 M Tris-HCl, pH 8.0). The DNA samples were then transferred to nylon membranes Hybond-XL; (Amersham Pharmacia Biotech, NJ, USA) and UV cross-linked in a Stratalinker 2400 (Stratagene). The HBV-specific probe was prepared with the Rediprime™ II Random Prime Labelling System (Amersham). The membranes were prehybridized at 65°C for 4 h in HYB-9 DNA hybridization solution (Gentra) and then hybridized with the ^32^P-radiolabeled DNA probe (2 × 10^8 ^cpm/μL). After hybridization for 16 h, the membranes were washed three times (20 min each) with 0.2 × SSC/0.1% SDS at 52°C and then exposed to X-ray film for 16 h at -80°C.

### Protein isolation and Western blot analysis

The cells were harvested for protein isolation in lysis buffer containing 1% Triton X-100 and the protease inhibitor Complete (Roche, Mannheim, Germany). The cell lysate was then centrifuged at 15, 500 × g for 15 min at 4°C, and the supernatant was collected. Total protein (100 μg) was separated by SDS-15% polyacrylamide gel electrophoresis (PAGE) and transferred onto PVDF membranes (Millipore Corp., Billerica, MA) using a Trans-Blot Semi-Dry Transfer Cell (Bio-Rad). The membranes were then blocked with 5% nonfat milk and probed with anti-HBc antibody (Dako Cytomation, Glostrup, Denmark) or anti-actin antibody (Sigma, St. Louis, MO). The immunoblot signals were detected with enhanced chemiluminescence reagent (PerkinElmer Life Sciences, Melbourne, Australia).

### HBV particle isolation for encapsidated viral genome analysis

The intracellular HBV core particles were analyzed as previously described [24,25]. Briefly, equal amounts of cell lysates were separated on a 1.2% native agarose gel and transferred onto nylon membrane to detect the capsid-associated nucleic acids. The capsid-associated nucleic acids were released from the core particles in situ by treating the membranes with 0.2 N NaOH/1.5 M NaCl and neutralizing them with 0.2 N Tris-HCl/1.5 M NaCl. Finally, the membranes were hybridized with HBV-specific probe.

### RNA preparation

The cells were washed twice with cold GKNP. TRIzol Reagent (Invitrogen) was then added, and the samples were placed on ice for 10 min to lyse the cells. For each 1 ml of TRIzol Reagent, 200 μl of chloroform was added and mixed by votexing for 15 s to homogenize the samples. The samples were centrifuged at 12, 000 rpm at 4°C for 15 min. Following centrifugation, the aqueous phase was transferred to a fresh tube, and the RNA was precipitated by adding 600 μl of isopropanol. The RNA pellets were collected after centrifugation and then dissolved in diethyl-pyrocarbonate-treated water. The RNA samples were ready for Northern blot analysis or reverse transcription (RT)-PCR.

### Northern blot analysis

Fifteen micrograms of total RNA were resolved electrophoretically on a 1.2% agarose gel with 2.2 M formaldehyde, followed by upward transfer to a nylon membrane overnight, and cross-linked under UV. In this experiment, the HBV-specific probe was prepared from the DNA fragment amplified by PCR using the primer pair HBV2338/F' and T20-Taq/HBV5. A hybridization probe for glyceraldehyde-3-phosphate dehydrogenase (GAPDH) was also prepared as the internal control for normalization. Prehybridization and hybridization were then performed in HYB-9 DNA hybridization solution (Gentra). After prehybridization at 65°C for 4 h, the probe was added and hybridized for 12-6 h. The membrane was washed three times with 1 × SSC/0.1% SDS buffer at 52°C for 20-30 min each. The RNA was detected by autoradiography with exposure to X-ray film for 16 h at -80°C.

### Southern blot analysis

Twenty micrograms of total DNA was digested with HindIII and separated on a 1.2% agarose gel. After electrophoresis, the agarose gel was soaked in denaturing buffer (0.5 M NaOH, 1.5 M NaCl) twice for 15 min each time and then neutralized with neutralizing buffer (10 M Tris-HCl [pH 8.0], 1.5 M NaCl). The DNA samples were then transferred to nylon membranes (Hybond-XL; Amersham Pharmacia Biotech) and UV cross-linked. The membranes were prehybridized at 42°C for 4 h in prehybridization solution and then hybridized in hybridization solution with a 32P-radiolabeled DNA probe (2 × 10^8 ^cpm/μl; prepared using random oligonucleotide priming of the whole HBV genome). After 16 h of hybridization, membranes were washed three times (20 min for each time) with 0.2× SSC and 0.1% SDS at 52°C and then exposed to X-ray film for 16 h at -80°C.

### Cell cycle analysis

Cells were harvested by trypsinization at each time course. The collected cells were then washed with PBS followed by 70% ethanol treatment. Finally the cells were incubated with 100 μg/ml RNaseA and 40 μg/ml propidium iodide (PI) for 30 minutes just before analysis by FACS calibur. The software Modfit LT was used to determine the cell cycle distribution after data acquisition.

### RT-PCR and restriction enzyme digestion

cDNA templates were generated by the reverse transcription of total mRNA with oligo(dT) 18-mer primer and SuperScript II reverse transcriptase (Invitrogen), and then amplified by PCR with the primer pair HBV2338/F' and T20-Taq/HBV5. The thermocycling parameters were 94°C for 1 min; five cycles of 94°C for 30 s, 50°C for 30 s, and 72°C for 30 s; 30 cycles of 94°C for 30 s, 55°C for 30 s, and 72°C for 30 s; and then a final extension at 72°C for 10 min. The PCR products were eluted from the agarose gel after electrophoresis and subjected to BclI/SspI double digestion. To determine the relative amounts of the RT-PCR products generated from the integrated genome or cccDNA, the restricted samples were separated again by electrophoresis on a 2% agarose gel.

### Quantification of HBsAg and HBeAg

The HBsAg and HBeAg in the culture medium were measured with an enzyme-linked immunosorbent assay (ELISA) Kit purchased from General Biology Corp The amount of formazan dye formed correlated with the amount of HBsAg or HBeAg, which was determined quantitatively using a scanning multi-well spectrophotometer (ELISA reader) at an absorbance of 450 nm. By using standards of HBsAg and HBeAg with known concentrations, the amounts of the secreted HBsAg and HBeAg were estimated.

### Quantification of the HBV genome

Viral DNA was extracted from the culture media with the High Pure Viral Nucleic Acid Kit (Roche, Mannheim, Germany) according to the manufacturer's instructions. A series of dilutions of known concentrations of HBV DNA was used as the control. The standard curve showed a good linear range when 10^2^-10^7 ^copies of plasmid DNA were used as the templates. The oligonucleotide sequences of the PCR primers were: HBV forward 5'-CAGGTCTGTGCCAAGT-3' and HBV reverse 5'-TGCGGGATAGGACAAC-3'. The PCR products were amplified using the SYBR Green PCR Master Mix (Roche). The PCR cycling program consisted of an initial denaturing step at 95°C for 10 min, followed by 45 amplification cycles at 95°C for 12 s and 54°C for 20 s.

### Quantitative real-time PCR

The Universal Probe Library was used to quantify the hepatocyte-specific markers and transcription factors. The relevant probes were selected from the Universal Probe Library Set (human), and the corresponding primers were combined with them. The primer pairs used in the experiment were: HLF-forward (5'-TTCATCCCTGATGACCTGAA-3'), HLF-reverse (5'-TGCCATGTTGTTCTTTCTGC-3'), and probe #61; PPARAα-forward (5'-GAAGCTGTCCTGGCTCAGAT-3'), PPARAα-reverse (5'-TCCCCGCAGATTCTACATT-3'), and probe #14; RXRα-forward (5'-ACATGCAGATGGACAAGACG-3'), RXRα-reverse (5'-GAGAGCCCCTTGGAGTCAG-3'), and probe #26; NR2F1-forward (5'-ATCGTGCTGTTCACGTCAGA-3'), NR2F1-reverse (5'-GCTCCTCACGTACTCCTCCA-3'), and probe #02; NFIL3-forward (5'-CTCCCCCACTACTGCAAGTC-3'), NFIL3-reverse (5'-ATCAGTTTCCGACGTTCTCG-3'), and probe #65; HNF3β-forward (5'-CGTTCCGGGTCTGAACTG-3'), HNF3β-reverse (5'-ACCGCTCCCAGCATACTTT-3'), and probe #68; HNF4α-forward (5'-AACCTGTTGCAGGAGATGCT-3'), HNF4α-reverse (5'-CGTTGGTTCCCATATGTTCC-3'), and probe #77; CEBPα-forward (5'-CAACACTTGTATCTGGCCTCTG-3'), CEBPα-reverse (5'-CGAGCAAAACCAAAACAAAAC-3'), and probe #03; HNF1α-forward (5'-TGAGTCCGGGCTTCACAC-3'), HNF1α-reverse (5'-GGCTGCTGGAGGACACTG-3'), and probe #42; Alb-forward (5'-ATGTTGCCAAGCTGCTGATA-3'), Alb-reverse (5'-CCTTCATCCCGAAGTTCATC-3'), and probe #27; G6P-forward (5'-GCTGCTCATTTTCCTCATCAA-3'), G6P-reverse (5'-TTCTGTAACAGCAATGCCTGA-3'), and probe #67; TAT-forward (5'-TGCTGAGCAGTCTGTCCACT-3'), TAT-reverse (5'-CTGCTCACAGAACTCCTGGAT-3'), and probe #67; and B_2_M-forward (5'-TTCTGGCCTGGAGGCTATC-3'), B_2_M-reverse (5'-TCAGGAAATTTGACTTTCCATTC-3'), and probe #42. The real-time PCR was performed using a Roche machine.

### Albumin secretion

The albumin secreted into the medium was measured with a Human Albumin ELISA Quantitation Kit from Bethyl Laboratories. Briefly, the culture medium collected at each time point was stored at -20°C until analysis. The samples were incubated on an ELISA plate coated with the capture antibody, followed by incubation with horseradish-peroxidase-conjugated antibody. Finally, the amount of albumin was measured with a microtiter plate reader at a wavelength of 450 nm by detecting the peroxidase activity after the substrate (TMB) had been added and the reaction stopped with H_2_SO_4_.

### Data analysis

Densitometric measurements of band intensities were made to quantify the proteins/nucleic acids with the AlphaEaseFC Imaging System software version 6.0.0 (Alpha Innotech Corp.). In all cases, the samples collected at the first time point were deemed to be 100%. All statistical analyses were performed with Student's *t *test.

## Results

### Dynamics of HBV replication during cell proliferation and growth confluency

**Figure 1:**
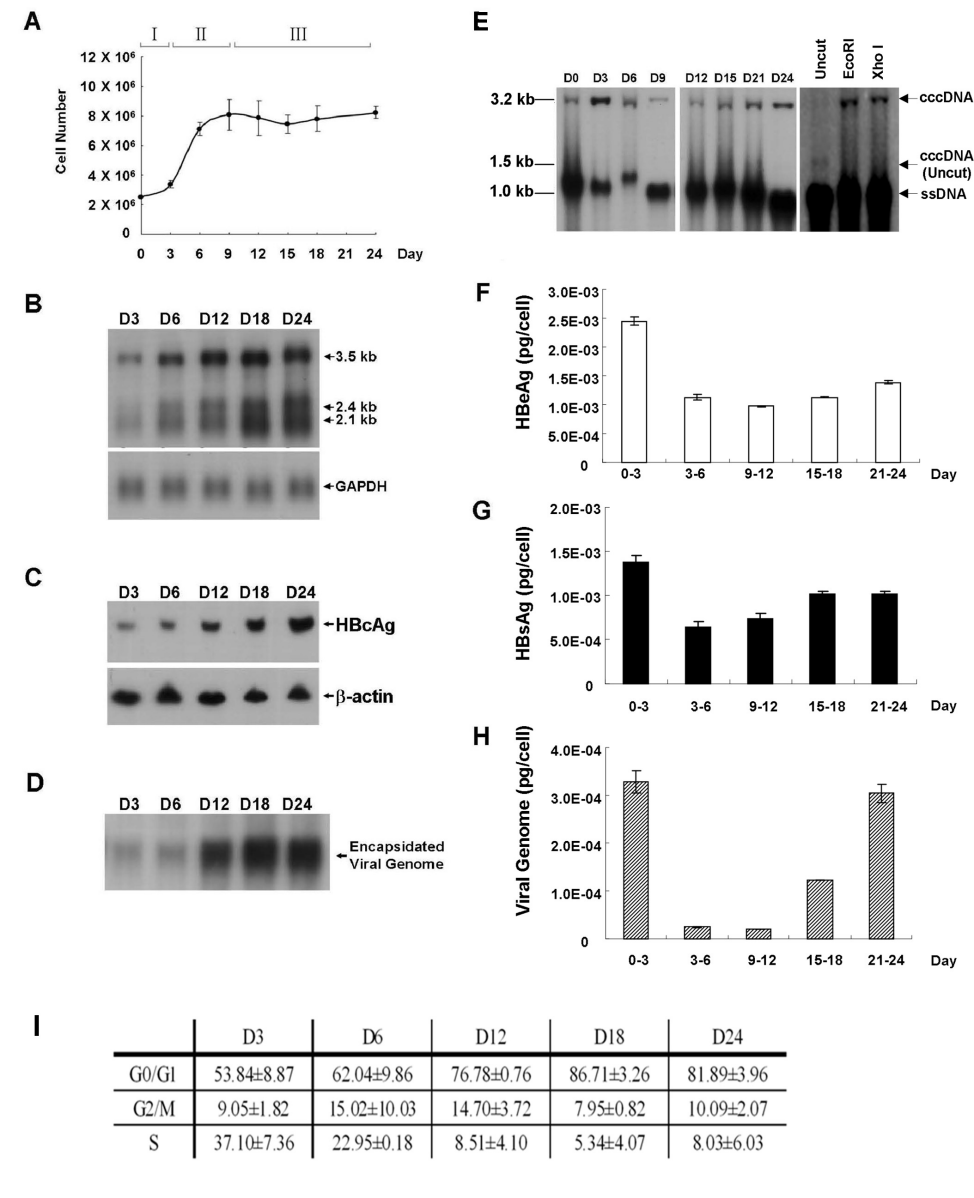
**HBV replication profiles in 1.3ES2 cells growing from proliferation to confluence**. 1.3ES2 cells (2.5 × 10^6^) were initially plated onto a 6 cm diameter Petri dish and cultured continuously for 24 days. The culture medium was refreshed every three days during the experimental period. (A) The cell number profiles were determined with the Trypan Blue exclusive method at the indicated time points. (B) Total RNA was extracted for Northern blot analysis and the expression profiles of HBV transcripts were determined. A GAPDH-specific probe was used to control for equal sample loading. (C) Cell lysates (100 μg) collected at the indicated time points were subjected to Western blot analysis for the detections of HBcAg and β-actin. (D) Equal amounts of cell lysates were subjected to native agarose gel electrophoresis for particle blot analysis. The encapsidated viral genomes were quantified by using an HBV-specific probe. (E) The intracellular cccDNA was collected by Hirt extraction at each time point. The cccDNA from equal amounts of cells was thermally denatured and then subjected to restriction enzyme digestion before electrophoresis and Southern blot analysis. The ssDNA represents the single-stranded viral DNA derived from the protein-free RC DNA by artificial heat denaturation. (F) The culture medium was collected at the indicated time points and the amounts of secreted HBeAg were determined by ELISA. (G) HBsAg production in the culture medium at each time point was analyzed by ELISA. (H) The secreted viruses were purified from the collected culture media, and the HBV titers were determined with quantitative RT-PCR analysis. (I) These growing cells were analyzed by FACS caliber at each time points and the cell cycle distributions were represented. Each time point represents the mean ± SD of triplicate experiments.

To explore the dynamics of HBV replication at different cell growth stages, the confluent 1.3ES2 cells (grown for 12 days) were re-plated to initiate cell growth and then continuously cultured for 24 days. The cell numbers and the HBV replication profiles were determined by three-day-intervals. According to the cell proliferation status, the cell growth curve was divided into three phases including (I) initial proliferation phase, (II) exponential proliferation phase, and (III) growth confluency phase (Figure 1A). To examine the correlation between HBV replication and cell growth stages more closely, a series of HBV replication categories, including intracellular viral events and secreted viral products, were analyzed (Figure 1B-H). A gradual increase of viral transcripts (Figure 1B) and intracellular HBc (Figure 1C) were observed in both proliferating cells (days 3-6) and confluent cells (days 12-24). Since the viral nucleocapsid is composed of pgRNA and viral core protein [5], we examined the formation of intracellular viral nucleocapsids in different cell growth stages by particle blot analysis (Figure 1D). The increased intensity of the encapsidated viral genome indicated the dramatic formation of intracellular viral nucleocapsids when cells were grown to confluence (Figure 1D, days 12-24). It has been well characterized that the intracellular viral nucleocapsids would either enter the nucleus to convert into viral cccDNA or secrete out of the cells [5,6]. Thus, we collected the Hirt extracts and culture media at various time points to analyze their viral contents (Figure 1E-H). After restriction enzyme digestion, the nature of viral cccDNA was revealed by the observation of band shift from 1.5 kb (uncut) to 3.2 kb (EcoRI or XhoI) (Figure 1E). Similar to the increase of intracellular viral particles in the confluent cells, the amount of cccDNA and the secreted viral products (HBe/cAg, HBsAg, and mature virion) were increased slowly as cells were grown to confluence (Figure 1E-H, days 9-24). However, the expression levels of cccDNA and secreted viral components in the proliferation stages were not coincidental with that of intracellular viral products. For example, the amount of cccDNA and the secreted viral products (HBe/cAg, HBsAg, and mature virion) were significantly elevated in the initial proliferation phase (Figure 1E-H, days 0-3) and then severely reduced in the exponential proliferation phase (Figure 1E-H, days 3-6). In contrast, the intracellular viral replications (viral transcripts, intracellular HBcAg, and intracellular viral capsids) were relatively less in the initial proliferation stage (Figure 1B-D, day 3) and were gradually increased during the exponential proliferation and the growth confluence stages (Figure 1B-D, days 6-24). To further clarify the HBV replication in different cell cycle phases of 1.3ES2 cells, the cell cycle distribution at indicated time points were analyzed (Figure 1I). By using FACS analysis, we dissected the cell populations of 1.3ES2 cells which were initially with 54% of G0/G1 phase and 37% of S phase at day 3. However, once reaching confluence, the cells were arrested at G0/G1 phase (77-87%) in together with reduction in S phase (5-9%) (Figure 1I, day 12-24). In recording the cell numbers by trypan blue exclusion method, we noticed that there was no significant increase in cell number after confluence (Figure 1A, day 12-24).

### The dynamic changes of HBV transcripts, intracellular viral DNA and cccDNA expression profiles during cell proliferation and growth confluency

**Figure 2:**
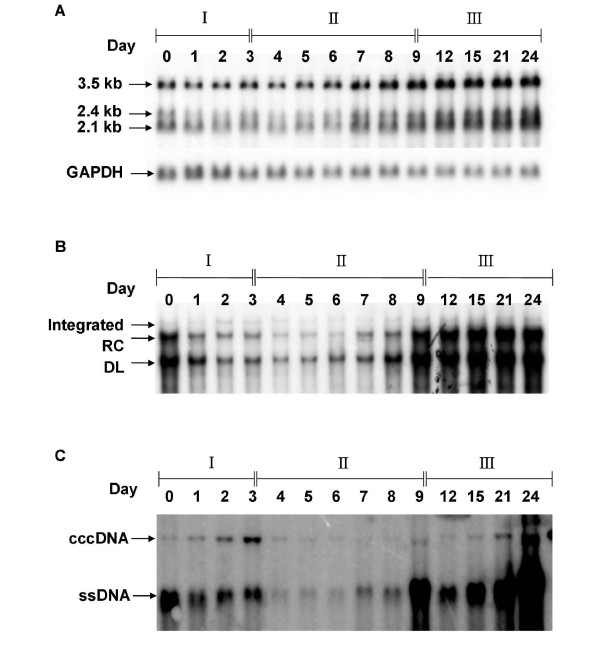
**The dynamic changes of HBV transcripts, intracellular viral DNA and cccDNA expression profiles in different cell growth phases**. 1.3ES2 cells (2.5 × 10^6^) were initially plated onto a 10 cm diameter Petri dish and cultured continuously for 24 days. The culture medium was refreshed every three days during the experimental period. (A) Total RNA was extracted at the indicated time points and subjected to Northern blot analysis to determine the expression profiles of the 3.5-kb, 2.4-kb, and 2.1-kb HBV transcripts. A GAPDH-specific probe was used to control for equal sample loading. (B) Total DNA was extracted at the indicated time points, digested with HindIII, and then separated by electrophoresis. The expression profiles of the integrated viral genome, RC DNA, and DL DNA were examined by Southern blot analysis. The integrated viral genome was used to control for equal sample loading. (C) The intracellular cccDNA was collected by Hirt extraction at each time point. The cccDNA was thermally denatured at 85°C for 5 min, and then subjected to *Eco*RI digestion before electrophoresis on a 1.2% agarose gel and Southern blot analysis.

Because the maintenance of HBV cccDNA in hepatocytes plays a key role in viral persistence and reactivation, it is important to understand the dynamics of HBV cccDNA expression in terms of cell growth stages. To obtain a clear picture how HBV replication affects cccDNA accumulation, the dynamics of HBV transcripts and viral DNA replicative intermediates were examined more precisely, especially during the initial proliferation phase and the exponential proliferation phase (Figure 2). The expression of HBV transcripts (3.5 kb, 2.4 kb and 2.1 kb) were gradually increased during cell proliferation and cell growth confluency (Figure 1B and 2A). In parallel to the slow increase of HBV transcripts, the expressions of viral relaxed-circular (RC) and duplex-linear (DL) form DNAs were significantly reduced in the initial proliferation phase, followed by gradually increased in the exponential proliferation phase and dramatically elevated in the growth confluency phase (Figure 2B). Since the RC DNA is the precursor of cccDNA, we next examined the expression profiles of cccDNA in different cell growth stages. As mentioned above, we found that there was a sudden increase in the cccDNA content of the cells during the initial proliferation phase (Figure 2C, days 0-3). When the cells underwent rapid proliferation in the exponential phase, the levels of cccDNA decreased significantly (Figure 2C, days 4-8). After that, the amount of cccDNA increased again gradually when the cells ceased to grow in the confluent phase (Figure 2C, days 9- 24). Interestingly, the expressions of ssDNA (single-stranded DNA derived from the protein-free DNA species by artificial heat denaturation) and intracellular viral DNA (RC DNA and DL DNA), were inversely proportional to the elevation of cccDNA in the initial proliferation phase (Figure 2B-C, days 0-3) but was strongly coordinated with the accumulation of cccDNA in the growth confluency phase (Figure 2B-C, days 9-24).

### Examination of the HBV cccDNA half-life in the HBV-producing 1.3ES2 cell line

**Figure 3:**
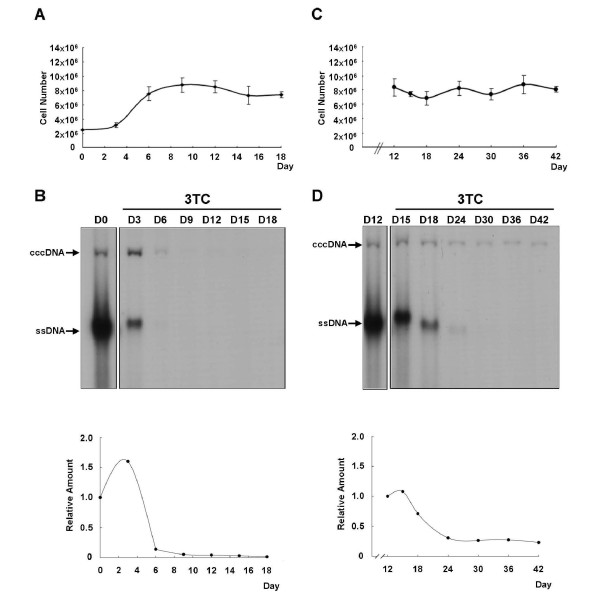
**Determination of the half-life of cccDNA in different growth stages**. (A) and (B) show the cell growth curve and cccDNA expression profile in the proliferating 1.3ES2 cells. The growth confluent cells were reseeded to initiate their proliferation, and 3TC was added at the day after plating. (A) The cell numbers were determined with the Trypan Blue exclusive method at the indicated time points. (B) The cccDNA before and after 3TC treatment was collected by Hirt extraction at each time point. The cccDNA was thermally denatured at 85°C for 5 min, then subjected to *Eco*RI digestion before electrophoresis on a 1.2% agarose gel and analyzed by Southern blot analysis. (C) and (D) show the cell growth curve and cccDNA expression profile in the confluent cells. After continuous culture for 12 days, the confluent cells were treated directly with 3TC from day 12 to day 42. (C) The cells numbers were determined with the Trypan Blue exclusive method at the indicated time points. (D) The Hirt extracts for each time point were thermally denatured at 85°C for 5 min, then subjected to *Eco*RI digestion before electrophoresis on a 1.2% agarose gel and analyzed by Southern blot analysis. The line charts represent the relative quantitative intensities of the cccDNA from each experiment.

Because cccDNA decreased rapidly during the exponential phase of cell growth (Figure 2C, days 4-8), we anticipated that rapid cell dividing may help to metabolize pre-existing cccDNA pool. To examine the half-life of HBV cccDNA in different cell growth phases, lamivudine (3TC) was used to inhibit HBV replication and to block the consequent de novo synthesis of cccDNA in 1.3ES2 cells. 3TC was added to the cells at two different time points after plating: one culture was treated with 3TC the day after the cells were reseeded, during the initial proliferation stage (Figure 3A-B), and another culture was treated with 3TC on day 12 after the cells were reseeded, when the cells were grown in the confluence phase (Figure 3C-D). Both groups of samples were collected every three days in the presence of 3TC, and the expression of cccDNA was measured by Southern blot analysis. The initial amounts of cccDNA in the two different groups were almost equal at the moment when 3TC was added (Fig, 3B, day 0; and Figure 3D, day 12), but their kinetics of cccDNA metabolism were quite different after prolonged 3TC treatment. We found that the half-lives of cccDNA measured in the rapidly growing cells and in the stationary cells were different. In the exponentially proliferating cells, the half-life of cccDNA was about five days, and it then became undetectable on Southern blots within nine days (Figure 3B). However, once the cells had grown to confluence, the half-life of cccDNA was about nine days, and the remaining 20% of the cccDNA was retained stably inside the cells for more than 30 days (Figure 3D). It suggests that the treatment of proliferating cells, but not growth confluent cells, with 3TC has the potential to remove cccDNA from HBV-producing cells.

### The evaluation of transcriptional efficiency of HBV cccDNA during cells growth from proliferation to confluency

**Figure 4:**
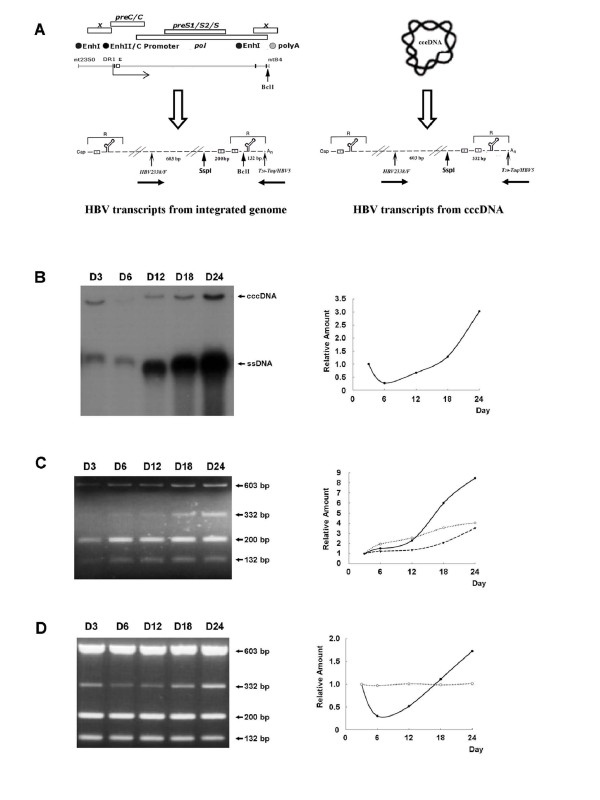
**Transcription profiles of HBV pgRNA during cell growth to confluence**. (A) Schematic representation of the strategy for detection of origins of viral transcripts. 1.3ES2 cells contain a 1.3-fold HBV genome with the *Bcl*I genetic marker in the 3' terminus redundancy. Viral transcripts from the integrated genome generate a *Bcl*I marker within the 3' terminus redundancy region, while transcripts derived from cccDNA does not. After RT-PCR (with primers HBV2338/F and T20-Taq/HBV5) and restriction enzyme digestion with *Ssp*I and *Bcl*I, the restricted PCR products are separated by agarose gel electrophoresis. The existence of 332-bp product, which lacks the *Bcl*I site, represents transcripts from cccDNA, whereas the two fragments of 200-bp and 132-bp represent transcripts from the integrated genome. (B) The cccDNA was collected with the Hirt extraction method at each time point, thermally denatured at 85°C for 5 min, and then subjected to *Eco*RI digestion before electrophoresis on a 1.2% agarose gel. All the lanes were loaded with samples representing the same numbers of cells. The autoradiograph shows the cccDNA and ssDNA; the line chart shows the relative quantitative intensities of the cccDNA. (C) Equal amounts of total RNA from each sample were used for RT-PCR with the primer pair HBV2338/F and T20-Taq/HBV5. Following *Ssp*I/*Bcl*I double digestion, the restricted PCR products were separated by electrophoresis. The relative quantitative intensities of the PCR products are shown in the line chart: 332-bp (--●--), 200-bp and 132-bp (...○...), and 603-bp (--▼--). (D) Equal amounts of the HBV RT-PCR products, amplified by the primer pair HBV2338/F and T20-Taq/HBV5, were subjected to *Ssp*I/*Bcl*I double digestion, and the restricted PCR products were separated by electrophoresis. The relative quantitative intensities of the PCR products are shown in the line chart: 332-bp (--●--), 200-bp and 132-bp (...○...).

Our previous studies had shown that 1.3ES2 cells contain a single integrated copy of the HBV genome and an average of 4-6 copies of cccDNA per cell at day 3, all of which can act as templates for viral transcription [23]. Because 1.3ES2 cells contain the *Bcl*I restriction site in the 3' terminal redundancy of the HBV genome, we can distinguish the two populations of viral transcripts simply by RT-PCR combined with *Ssp*I/*Bcl*I digestion [23]. Briefly, the HBV transcripts were first amplified by RT-PCR with an HBV-specific primer pair (HBV2338/F and T20-Taq/HBV5), and the 935-bp PCR product was then subjected to *Ssp*I/*Bcl*I double digestion. The resulting amounts of the 132-bp and 200-bp DNA fragments were proportional to the amount of mRNA derived from the integrated genome. In contrast, the amount of the 332-bp PCR fragment after *Ssp*I/*Bcl*I double digestion was proportional to the mRNA derived from cccDNA (Figure 4A). Equal amounts of total RNA extracted at different time points were analyzed by RT-PCR and subsequent *Ssp*I/*Bcl*I double digestion (Figure 4C). As the cells grew to confluence, the transcripts from the cccDNA (332-bp fragment) and from the integrated viral genome (132-bp and 200-bp fragments) both gradually increased (Figure 4C), which is consistent with our previous observations with Northern blot analysis (Figure 1B and 2A). Even though the transcripts from cccDNA were relatively less, the elevation of transcripts from cccDNA was significantly more efficient than that from the integrated viral genome (Figure 4C, right panel). To further confirm our observation, the proportions of HBV transcripts from the two transcriptional origins were monitored by using equal amounts of HBV RT-PCR products (as demonstrated by the same intensity of 603-bp signals) (Figure 4D). The proportion of transcripts from the cccDNA was lower than that from the integrated genome, which accounted for less than 10% of the total viral transcripts on day 6 (Figure 4D). However, the proportion of transcripts from the cccDNA was increased significantly once the cells reached confluence (Figure 4D, days 12-24). Interestingly, the increase in transcripts derived from the cccDNA was proportional to the increase of cccDNA during cell growth from proliferation to confluence (Figure 4B and 4D, right panels). Meanwhile, the proportion of transcripts from the integrated viral genome remained almost constant during cell growth (Figure 4D, right panel).

### Expression of transcription factors and differentiation markers in long-term cultures of stably HBV-producing cells

**Figure 5:**
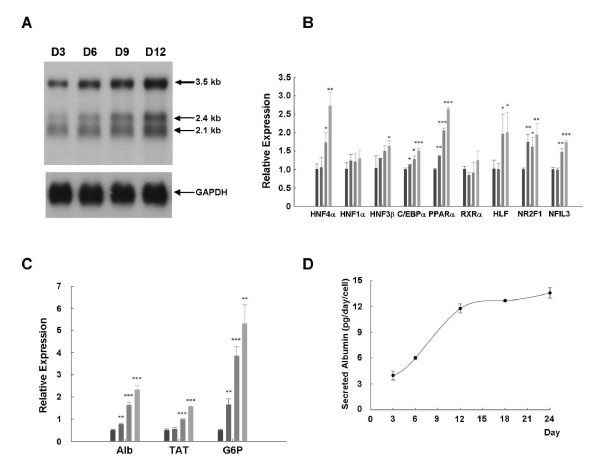
**Expression levels of transcription factors associated with the regulation of HBV transcription**. (A) Total RNA was extracted at the same time points and subjected to Northern blot analysis to determine the expression profiles of the HBV transcripts: the 3.5-kb, 2.4-kb, and 2.1-kb transcripts (upper panel). The amount of sample loaded was normalized with a GAPDH-specific probe (lower panel). (B) The expression of transcription factors that regulate HBV pgRNA transcription was assessed by quantitative real-time RT-PCR at the indicated time points. The bar chart shows the relative expression of HNF4α, HNF1α, HNF3β, CEBPα, PPARα, RXRα, HLF, NR2F1, and NFIL3 mRNAs which was normalized to that of B2M. ■ day 3 sample; ■ day 6 sample; ■ day 9 sample; ■ day 12 sample. * *P *< 0.05, ** *P *< 0.01, and *** *P *< 0.001. The data were analyzed statistically with Student's *t *test. (C) The expression profiles of hepatocyte-specific markers (albumin, TAT, G6P mRNAs) were determined by real-time RT-PCR. The results are shown after normalization to B2M. ■ day 3 sample; ■ day 6 sample; ■ day 9 sample; ■ day 12 sample. (D) The culture media were collected at the indicated time points, and the amounts of secreted albumin were determined by ELISA.

Despite the constant amount of integrated viral genome within the cells, the transcripts from the integrated HBV genome (132-bp and 200-bp fragments) increased gradually during cell growth to confluence (Figure 4C). This indicates that the transcription efficiency of the HBV transcripts from the integrated viral genome is enhanced during cell growth from proliferation to confluency. Therefore, we next examined the expression profiles of hepatic transcription factors during long-term cell growth. Because many cellular transcription factors are reported to regulate HBV transcription [26], the expression profiles of these transcription factors in continuously cultured cells were investigated. Total RNA was extracted at various time points, and the expression profiles of the viral transcripts during cell growth were examined for reference (Figure 5A). Quantitative RT-PCR analysis demonstrated that the expressions of several well-known positive regulators of HBV transcription, including HNF4α, CEBPα, PPARα and HLF, were elevated in long term cultured cells (Figure 5B). Simultaneously, the expression of HNF1α and RXRα remained unchanged during that period. These changes in the expression pattern of hepatic transcription factors might contribute, at least in part, to the consequence of transcriptional up-regulation of HBV replication during cell growth to confluence. However, we do not exclude the possibility that such an expression profile changes of these transcription factors during cell growth might be specific to the cell line we used.

It has been suggested that the expression of these hepatic transcription factors coordinately regulates hepatocyte differentiation [27-30] and that cellular differentiation is usually preceded by cell growth arrest [19,31-33]. Therefore, we analyzed the expression of hepatocyte-specific differentiation markers by quantitative RT-PCR and examined their correlation with cell growth phases. The expression of albumin, tyrosine aminotransferase (TAT), and glucose-6-phosphatase (G6P) was up-regulated when the cells became confluent (Figure 5C). The culture medium was collected to determine the cellular albumin secretion. Consistent with the elevated levels of albumin transcriptions, the amount of secreted albumin increased progressively during cell growth to confluence (Figure 5D).

## Discussion

### Dynamics of HBV replication in three different cell growth phases

**Figure 6:**
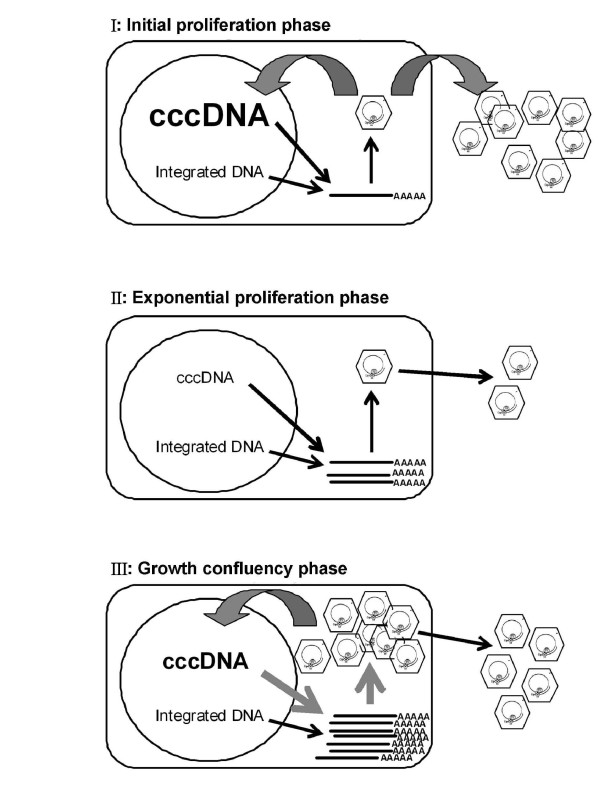
**Illustration of the three growth-stage model ofr HBV replication in 1.3ES2 cells**. The growth curve of HBV can be separated into three phases: (I) the initial proliferation phase; (II) the exponential proliferation phase; and (III) the growth confluency phase. In the initial proliferation phase, the increase in cccDNA replenishment in the nucleus during the initial cellular proliferation generates a dramatic increase in HBV cccDNA inside the cells. The increased secretion of HBV capsids and low levels of cellular transcription factors explain the relative inactive HBV replication and the inefficient viral transcription inside the cells. In the exponential proliferation phase, the cccDNA decreases significantly, probably because of the dilution effect caused by cell division. The viral transcripts and intracellular nucleocapsids gradually increase in this stage. In the growth confluency phase, HBV transcription dramatically elevates with the increase in cellular transcription factors as the cells reach confluence. Meanwhile, the gradual accumulation of cccDNA and intracellular viral particles, together with elevated viral secretion are observed in these confluent cells.

In summary, we present here a three-phase model to describe the different phenomena involved in HBV replication (Figure 6). Based on the proliferation statuses of HBV-producing cells, we discuss the replication of HBV by three different cell growth phases: (I) initial proliferation phase, (II) exponential proliferation phase, and (III) growth confluency phase (Figure 2). We found that the dynamics of HBV transcripts expression, intracellular viral DNA (RC and DL DNA), and cccDNA profiles were quite different in these three growth phases. For example, the amount of HBV transcripts was relatively low in both the initial and exponential proliferation phases, but was significantly increased when the cells ceased to grow during confluence (Figure 2A). On the other hand, the amount of intracellular viral DNA was relatively high at the beginning of proliferation (Figure 2B, day 0), and then significantly decreased in the initial proliferation phase (Figure 2B days 0-3). After that, the levels of viral RC and DL DNA were gradually increased during exponential proliferation phase (Figure 2B, days 4-8) and dramatically accumulated in the cell growth confluency phase (Figure 2B, days 9-24). In contrast, a dramatic increase of HBV cccDNA pool was observed in the end of initial proliferation phase (Figure 2C, day 3), followed by a severe drop in cccDNA amount once cells were exponentially growing (Figure 2C, day 4). The amount of cccDNA maintained at a relatively lower level in the exponential proliferation stage and then accumulated progressively after growth confluency. The detail mechanisms of these inconsistent phenomena were further discussed below.

### The correlation between cell cycle phases and cccDNA accumulation

There are two previous achieves on studying the relationship between cell cycle phases and cccDNA accumulation [34,35]. By using different cell cycle blocker (Aphidicolin or n-Butyrate), both groups examined the expression level of cccDNA in the cells of G1 arrest, but their studies led to completely conflicting conclusions. The inconsistency might caused by their different model systems (HBV-produced HepG2 cell line vs. In Vitro infection of duck hepatocyte cultures), using different G1 blockers (Aphidicolin vs. n-Butyrate), or different experimental designs used by these two groups. Our results showed that the accumulation of cccDNA was parallel with G1 arrest when cells were grown to conflurence (Figure 1A, E, and 1I), which is consistent with the previous report done by Yeh CT et al. [34]. They demonstrated that viral RC-DNA imported into nuclear at the G1 phase and cccDNA accumulated after prolonged treatment of Aphidicolin (a G1 blocker). Different from their strategy, we kept culturing 1.3ES2 cells for 12 days (cell growth confluence) before re-plating instead of synchronization of these cells by serum starvation. Thus, large amounts of RC-DNA accumulated by 12 days of culture would serve as the source of cccDNA, once cells begin their initial proliferation after re-plating. However, our results also showed that the increase of cccDNA pool in the initial proliferation stage was not correlated to its G1 phase. This conflicting result indicated that the accumulation of cccDNA is not simply laid on the cell cycle phase (such as G1 arrest) and other parameter might also involve in the modulation of cccDNA pool. For example, as observed in the initial proliferation stage, large amount of protein-free RC-DNA and slower cell dividing rate might contribute, at least in part, to its conversion of RC-DNA to cccDNA. Consequently, we observed that the increase of cccDNA was accompanied with the decrease of protein-free viral DNA (ssDNA) in this stage (from day 0 to day 3). Moreover, we found that the cccDNA pool was dramatically reduced in the exponential proliferation stage (from day 3 to day 9), which is consistent with the findings (by Yeh CT et al. [34]) that a rapidly growing cell culture possibly did not have a G1 phase long enough for supporting the conversion of RC-DNA to cccDNA.

### The dynamic expression of HBV transcripts and its regulation in different cell growth phase

As mentioned before, both integrated HBV genome and cccDNA molecule are able to serve as origins of HBV transcripts [23]. However, the integrated HBV genome and the dynamic changes in cccDNA amount could only partially explain the gradual elevation of viral transcripts during cell growth. For example, in the growth confluency phase, cccDNA progressively accumulated inside the nucleus, providing increased templates for the transcription of viral mRNA. Indeed, the proportion of transcripts from cccDNA perfectly matched the kinetics of cccDNA accumulation during cell growth (Figure 4B and 4D, right panels). Nevertheless, the amount of cccDNA is not the only parameter involved in the elevation of viral mRNA expression. Our study revealed that the elevation of hepatic transcription factors might also contribute to the activation of HBV transcription. In the initial proliferating cells, we observed that the relatively lower amount of hepatic transcription factors paralleled the inefficient synthesis of viral transcripts even with a large amount of cccDNA in this stage (Figure 2A, C and 5B, day 3). In contrast, the expression levels of several hepatic transcription factors were elevated in the growth confluency phase (Figure 5B). Among these, HNF4α, PPARα, C/EBPα, and HLF have been suggested to function as positive regulators of HBV replication. For example, HNF4α and RXRα/PPARα have been shown to support HBV pgRNA synthesis and viral biosynthesis in nonhepatic cells [26,36]. C/EBPα, together with HBx, has been reported to enhance the HBV pregenomic promoter synergistically in hepatocytes [37]. HLF also exerts a stimulatory effect on the HBV promoter and strongly stimulates the synthesis of pgRNA [38]. Taken together, these elevated hepatic transcription factors might coordinate with the accumulated cccDNA and comprehensively enhance HBV transcription efficiency as the cells gradually ceased to grow (Figure 4C, right panel). In conclusion, our data suggest that the transcriptional regulation of HBV is tightly controlled by the dynamic changes in cccDNA and cellular transcription factors during cell growth. Moreover, our results also showed the coordinately enhanced differentiation of 1.3ES2 cells as cells were grown to confluence (Figure 5C-D). The differentiation status of the hepatocytes paralleled the elevation of several hepatocyte-enriched transcription factors, which might stimulate viral transcription and enhance HBV replication. These findings were coincident with previous reports which showed a positive correlation between cell differentiation status and viral replication [39,40].

### Kinetic expression of intracellular DNA in different cell growth phases

The high intensity of viral RC and DL DNA in the beginning of initial proliferation stage indicated that large amount of intracellular nucleocapsids was accumulated before cell re-plating. However, once these cells started to proliferate, the intensity of intracellular viral DNA was gradually decreased (Figure 2B, days 0-3). Similar phenomena were found by tracing the dynamic expression of protein-free viral DNA (ssDNA), the presumed precursor of cccDNA [41] (Figure 2C, days 0-3). Meanwhile, a dramatic increase of HBV cccDNA pool was observed (Figure 2C, days 0-3), which suggested that the intracellular nucleocapsids were replenished into the nucleus and the embedded viral DNAs were converted to cccDNA in this stage. Coincident with the significant increase of HBV transcripts, the intracellular nucleocapsids (as revealed by the signals of RC and DL DNA) were dramatically elevated when these cells were grown to confluence (Figure 2B, days 9-24).

### Dynamic expression of cccDNA in different cell growth phases

As mentioned above, the sudden increase of cccDNA was accompanied with a reduction of intracellular nucleocapsids when the host cells underwent their initial proliferation (Figure 2B-C, days 0-3). It seems that the disappearance of the nuclear membrane during mitosis facilitated the entry of pre-existing intracellular nucleocapsids into the nucleus, and the released protein-free RC DNA was consequently converted to cccDNA in the initial proliferation stage. However, once cells entered the exponential phase, the amount of cccDNA was declined dramatically (Figure 2C, days 4-8). Because the cccDNA lacks a replication origin, the reduction of cccDNA in the rapidly proliferating cells might be caused by a dilution effect similar to the loss of extrachromosomal plasmid DNA during cell division [42,43]. After the cells reached confluence and ceased to grow, the intracellular nucleocapsids and protein-free viral DNA (ssDNA) accumulated markedly (Figure 2B-C, days 9-24), which could account for the gradual replenishment of cccDNA in the growth confluency phase. This observation is consistent with the previous report in which the HBV cccDNA formation in cultured cells is accompanied by the accumulation of protein-free viral DNA species [41].

### The evaluation of viral transcriptional efficiency during cells growth to confluence

To evaluate the transcriptional efficiency of cccDNA in different cell growth phase, we carried out the RT-PCR combined with the restriction enzyme digestion assay [23]. We found that the transcriptional ability of cccDNA was less efficient then that from integrated viral genome in all cell growth stages (Figure 4C, left panel). By comparing the relative intensities of PCR fragment from cccDNA (332-bp) and these from integrated viral genome (200-bp and 132-bp), the estimated proportion of transcripts from cccDNA relative to the amount from the integrated HBV genome was only 5% to 20% (Figure 4D and data not shown). However, the detail mechanism how transcription regulators manipulate the differential transcription efficiencies of cccDNA and integrated viral genome is not clarified yet. In spite of the lower amount of transcripts from cccDNA, the elevating potency of transcription from cccDNA was significantly raised during cell growth as compared with that from integrated viral genome (Figure 4C, right panel). To clarify precisely the proportions of transcripts from these two origins during cells growth to confluence, equal amounts of RT-PCR products were used to examine their transcriptional origins (Figure 4D). Interestingly, the proportional change of transcripts from cccDNA (Figure 4D, right panel) completely paralleled with the dynamic expression of cccDNA during cell growth (Figure 4B, right panel). In the meanwhile the proportion of transcripts from integrated genome remained constant (Figure 4D, right panel). It indicates that the higher elevation potency of transcription by cccDNA was resulted from the increase of cccDNA pool within the cells when cells were grown to confluence.

### Modulation of cccDNA metabolism by cell proliferation

The amount of cccDNA was estimated to be approximately 4-6 copies of HBV cccDNA per cell on day 3 [23]. During exponential proliferation, the cccDNA decreased rapidly to less than 0.4-0.6 copies per cell. Thereafter, the number of cccDNA copies per cell accumulated continuously during cell growth to confluence (Figure 4B). The constant presence of cccDNA in the hepatocyte is generally considered to play critical roles in the HBV life cycle, including in viral replication, persistent infection, and recurrence. cccDNA also functions as a risk marker for the development of hepatocellular carcinoma because hepatocarcinoma tissue is reported to have higher levels of cccDNA than non-tumor tissues [44]. Therefore, the eradication of cccDNA from infected hepatocytes is considered to be the therapeutic goal for viral clearance in CHB patients. With lamivudine (3TC) treatment, we blocked the formation of newly synthesized cccDNA and determined the half-life of cccDNA in both proliferating cells and growth confluent cells (Figure 3). We found that the cccDNA molecule is extremely stable in the confluent cells, which still retained a constant amount of cccDNA after prolonged treatment with 3TC for 30 days (Figure 3D). This observed longevity of cccDNA is consistent with previous reports that cccDNA is a very stable molecule [10,11]. Surprisingly, we found that cccDNA was dramatically reduced in the proliferating cells (Figure 3B). Without replenishment of cccDNA, the pre-existing cccDNA pool was dramatically diluted by rapid cell division. Moreover, the trace of cccDNA became undetectable by treating proliferating cells with 3TC for 9 days (Figure 3B). It suggests that the half-life of cccDNA is reduced and that the molecule is relatively unstable in proliferating cells. Several lines of evidence confirm our finding that cell division efficiently dilutes the amount of cccDNA in infected hepatocytes. For instance, in congenitally DHBV-infected hepatocyte cultures, the half-life of the DHBV cccDNA was estimated to be only 3-5 days in continuously proliferating cells, which confirms the loss of cccDNA during cell division [45]. Similarly, a kinetic study of WHV loss after treatment with an antiviral agent suggested that the reduction in cccDNA might have been caused by its redistribution to the daughter cells of the infected hepatocytes [10]. Moreover, an in vitro proliferation study of HBV-infected hepatocytes showed that cell division plays a crucial role in cccDNA clearance in the chronically infected hepatocytes [16]. Because the elimination of cccDNA is essential for HBV clearance, our study provides the notion that the combination of treatment with an antiviral agent and the induction of cell proliferation, such as limited inflammation or partial hepatectomy, might accelerate cccDNA metabolism and facilitate viral clearance.

## Conclusions

In this study, the dynamic changes of HBV cccDNA, viral transcripts, and viral replication intermediates in different cell growth stages were well investigated. Our study elucidated how the growth status of hepatocytes affects the formation of cccDNA. A three-phase scenario (initial proliferation phase, exponential proliferation phase, and growth confluency phase) describing the dynamic expression of viral cccDNA and its association with HBV replication was proposed. Moreover, we found that the half-life of cccDNA is reduced in dividing cells treated with lamivudine (3TC), and the cccDNA molecule could be completely removed by long-term treatment of 3TC. The significance of our findings regarding HBV regulation in CHB patients was discussed.

## List of abbreviations

cccDNA: covalently closed-circular DNA; HBV: hepatitis B virus; CHB: chronic hepatitis B; pgRNA: pregenomic RNA; CTL: cytotoxic T-lymphocyte; 3TC: lamivudine; TAT: tyrosine aminotransferase; G6P: glucose-6-phosphatase.

## Competing interests

The authors declare that they have no competing interests.

## Authors' contributions

CLC performed the major experiments and analyzed the data. CPH, MLC and CC participated in the design of the study and data interpretation. YCW, KNT and CCH participated in part of the experiments. KSJ established the 1.3ES2 cell line. YCC and CC designed the experiments, interpreted the data and wrote the manuscript. All authors read and approved the final manuscript.
